# Effect of Age in Auditory Go/No-Go Tasks: A Magnetoencephalographic Study

**DOI:** 10.3390/brainsci10100667

**Published:** 2020-09-25

**Authors:** Mei-Yin Lin, Chia-Hsiung Cheng

**Affiliations:** 1 Department of Occupational Therapy and Graduate Institute of Behavioral Sciences, Chang Gung University, Taoyuan 333, Taiwan; meiqu5210@gmail.com; 2 Laboratory of Brain Imaging and Neural Dynamics (BIND Lab), Chang Gung University, Taoyuan 333, Taiwan; 3 Department of Physical Medicine and Rehabilitation, Taichung Hospital, Ministry of Health and Welfare, Taichung 403, Taiwan; 4 Healthy Aging Research Center, Chang Gung University, Taoyuan 333, Taiwan; 5 Department of Psychiatry, Chang Gung Memorial Hospital, Linkou 105, Taiwan

**Keywords:** inhibition, aging, magnetoencephalography (MEG), auditory go/no-go

## Abstract

Response inhibition is frequently examined using visual go/no-go tasks. Recently, the auditory go/no-go paradigm has been also applied to several clinical and aging populations. However, age-related changes in the neural underpinnings of auditory go/no-go tasks are yet to be elucidated. We used magnetoencephalography combined with distributed source imaging methods to examine age-associated changes in neural responses to auditory no-go stimuli. Additionally, we compared the performance of high- and low-performing older adults to explore differences in cortical activation. Behavioral performance in terms of response inhibition was similar in younger and older adult groups. Relative to the younger adults, the older adults exhibited reduced cortical activation in the superior and middle temporal gyrus. However, we did not find any significant differences in cortical activation between the high- and low-performing older adults. Our results therefore support the hypothesis that inhibition is reduced during aging. The variation in cognitive performance among older adults confirms the need for further study on the underlying mechanisms of inhibition.

## 1. Introduction

Due to the increasing prevalence of ageing populations over the last few decades, the concern about cognitive decline in older adults has increased [1]. Previous studies have proposed that inhibitory dysfunction is a major cause of cognitive decline in older age [2,3,4], whereby older adults may experience mental interference from irrelevant contextual details and are unable to suppress prepotent actions that are no longer required [5,6,7]. Effective social interaction requires functional prospective memory, naming, and speech production, which may deteriorate in older adults due to defects in inhibitory function [8,9,10,11]. This cognitive decline affects older adults’ well-being and quality of life [12]. Therefore, response inhibition is essential for achieving successful cognitive and motor control in a constantly changing environment [13].

The restraint component of inhibitory processing has been studied extensively using go/no-go tasks [14]. Previous electrophysiological studies have reported N2/P3 event-related potentials in the 200–600 ms time window to be the most prominent component revealing the top-down control process in go/no-go tasks [15,16,17]. The age-associated changes in cortical activation underlying go/no-go tasks have primarily been explored using the visual modality. There is accumulating evidence indicating that older adults perform more poorly than younger adults [18,19,20,21,22,23]. In our previous visual go/no-go study, the older adult group exhibited reduced cortical activation in the middle temporal gyrus (MTG) and delayed latencies in the MTG, prefrontal cortex (PFC), and pre-supplementary motor area (pre-SMA) [22]. However, the activation of precise brain regions during auditory go/no-go tasks in older individuals is yet to be elucidated.

Auditory go/no-go tasks have been used in several clinical populations, including subjects with schizophrenia [24], Down syndrome [25], attention-deficit hyperactivity disorder [26], or borderline personality disorder [27]. In addition, several studies have explored age-related changes using behavioral measures and electroencephalographic (EEG) recordings. Using these methods, no significant differences in behavioral performance relating to response accuracy were found between younger and older adults; however, reaction time (RT) was longer in older than younger adults [18,28,29]. Based on EEG recordings, older adults were found to exhibit smaller recruitments and exhibit longer latencies in neural activation than younger adults [18,29]. Older adults have also been found to demonstrate more activity in frontal regions of the brain during auditory go/no-go tasks [29], which is congruent with previous findings based on visual tasks [30,31,32]. However, the neural correlates of auditory response inhibition remain to be elucidated. Several studies have proposed that response inhibition is a common underlying process in both modalities [33,34], although whether it is similarly affected by aging in both modalities is debated [16,18]. For instance, Seli and colleagues reported strong correlations between visual and auditory modalities, but noted that auditory tasks were easier to handle with vulnerable populations [34]. In other words, age-related changes in the inhibition process might be differentially susceptible across sensory modalities. It is therefore important to explore the spatiotemporal characteristics of age-related cortical changes in auditory go/no-go tasks.

Magnetoencephalography (MEG) can detect neural activation in the millimeter range with less distortion by head tissues more accurately than EEG, thus providing a finer spatial resolution [35,36]. To date, only one MEG study has used auditory go/no-go tasks to examine error processing in incorrect no-go trials, and no studies have yet examined cortical activation during inhibitory processes [37]. Our previous study, based on the visual go/no-go paradigm, found that among older participants, high performers exhibited greater left PFC activation than low performers [22]. Several studies have found greater variation in cognitive performance among older individuals [38,39,40]. However, differences in cortical activation between high- and low-performing older adults during response inhibition have not been well investigated using other sensory modalities, such as auditory stimuli. Since the inhibitory function is also thought to vary greatly among older adults [41], we aimed to assess differences in neural activation between high- and low-performing older adults based on response inhibition (i.e., false alarm (FA) rate) in auditory go/no-go tasks.

This study had two specific aims. Our first aim was to examine age-related changes in cortical activation using the auditory go/no-go paradigm, based on MEG recordings. Our second aim was to explore the cortical activation differences in response to successful no-go stimuli between high-performing and low-performing older adults. Based on findings in the literature, we hypothesized that older adults would exhibit a similar response accuracy but longer reaction time than younger adults. Cortical responses would be smaller in strength with longer latencies in the older adult group. Moreover, we also expected to find differences in cortical activation patterns between high- and low-performing older adults in auditory go/no-go tasks, consistent with our previous results based on the visual modality.

## 2. Materials and Methods

### 2.1. Subjects

We recruited 20 younger adults (10 of whom were females; mean age 24.4 years) and 20 healthy community-dwelling older adults (12 of whom were females; mean age 62.6 years) as participants in this study. The majority of participants were sampled from our previous investigation [22]. Based on a self-report, all participants were right-handed; had no history of neurological or psychological disorders; had normal hearing, and had normal or corrected-to-normal (with contact lenses) vision. None of the older adults were at risk of dementia based on the Cognitive Abilities Screening Instrument [42] for which all participants had a score of >90/100. This study was conducted in accordance with the Declaration of Helsinki and approved by the Institutional Review Board of Taipei Veterans General Hospital (2014-12-005CC). All participants provided written informed consent.

### 2.2. MEG Paradigm and Experimental Design

The auditory stimuli were spoken digits from one to nine, which were delivered via earphones with an intensity of 70–80 dB to ensure that the participants could hear them clearly. These spoken digits were synthesized using the Industrial Technology Research Institute’s Text-To-Speech Web Services (http://tts.itri.org.tw/). The duration of each stimulus was 250 ms, and the inter-stimulus interval (ISI) varied between 1400 and 2200 ms to avoid anticipation effects. The go stimuli were all digits except 3 and 7, which were the no-go stimuli.

During the MEG recordings, participants were instructed to look at a white crosshair in the center of a black screen throughout the experiment. During the go/no-go task, they were asked to respond as quickly and correctly as possible to the go stimuli (i.e., 1, 2, 4, 5, 6, 8, or 9) and not to respond to the no-go stimuli (i.e., 3 and 7). Responding involved moving their right index fingers, which were attached to a small plate with a light-emitting diode sensor to record their behavioral responses. After 20–25 practice trials, two blocks with a total number of 450 trials were conducted, in which 315 (70%) go and 135 (30%) no-go stimuli were presented.

### 2.3. Behavioral Assessments

We measured reaction time (RT), miss rate, and false alarm (FA) rate as indices of behavioral performance. The go stimuli RT recordings excluded responses that were too fast (<200 ms following stimulus onset) or too slow (>1000 ms). We used the miss rate as an index of the subjects’ attention levels during the experiment. It was defined as the number of missed go stimuli divided by the total number of go trials, and a higher miss rate indicated a greater attention deficit. The FA rate was used as an index of disinhibition and was defined as the ratio of incorrect no-go responses to the number of no-go trials. A higher FA rate indicated a lower level of the inhibitory function involved in restraining unnecessary responses [14,43].

### 2.4. MEG Recordings and Pre-Processing

Neuromagnetic signals were acquired using a whole-scalp 306-channel MEG device (Vectorview, Elekta-Neuromag, Helsinki, Finland). Before conducting the MEG recordings, we marked four indicator coils using a three-dimensional digitizer to link with the MEG sensors, thus capturing the precise location of the head. Second, three anatomical fiducial points (nasion and bilateral preauricular points) and additional head points were uniformly marked on the surface of the scalp. These digitized head points assisted with connecting the MEG file with the default anatomy. The MEG data were collected by 204 planar gradiometers, which detected the maximal evoked brain activity and minimized external magnetic disturbances [44].

We collected at least 70 artifact-free, correct no-go trials from each participant. The raw data were digitized at 600 Hz and the recording bandpass was set at 0.1–200 Hz. Subsequent offline bandpass processing was set at 1–40 Hz to avoid contamination by muscle artifacts [45,46]. Eyeblink artifacts were removed using signal space projections [47] in the Brainstorm software [48]. In brief, this method applied principle component analysis on the concatenated artifacts to obtain a decomposition into various spatial components. Following the selection of components, linear projectors were computed for each spatial component to remove identified artifacts and then applied to the MEG data.

### 2.5. Data Analysis and Statistics

#### 2.5.1. Behavioral Data

Statistics are reported as mean ± standard error of the mean (SEM). The behavioral performance indicators (RT, miss rate, and FA rate) were compared using independent samples *t*-tests to test for age-related differences. We divided the older adults into two equal-sized subgroups based on their FA rate. These were the high- and low-performing groups, and we compared the differences between them in behavioral performance using independent samples *t*-tests.

#### 2.5.2. MEG Data

We used an overlapping sphere model to address source estimation of the electrical dipoles from the scalp surface [49]. The magnetic responses were analyzed using a depth-weighted minimum norm estimate (MNE) approach. It has been mathematically shown that the L2-MNE of neural currents can be used to localize distributed neural activation [50,51]. Individual brain networks were constructed using the Brainstorm software registration measures (i.e., cortically constrained MNE) to map the source space, which consisted of 15,000 elementary current dipoles. The MNE cortical currents of each participant were averaged onto the adjusted ICBM152 brain template which fitted the digitized head points on the individual scalp surface [52].

We corrected the baseline noise of each dipole based on the pre-stimulus interval from −100 to 0 ms and subsequently normalized the event-related magnetic signals to *z*-score estimates to increase statistical reliability [53]. Absolute *z*-scores of magnetic signals were extracted for statistical analysis. Based on the grand-averaged source waveforms, we recorded peak amplitude and peak latency of the magnetic signals in the 200–600 ms (N2/P3) time window following no-go stimulus onset, which corresponds to the temporal processing window of response inhibition in the brain [14,17,54].

**Figure 1:**
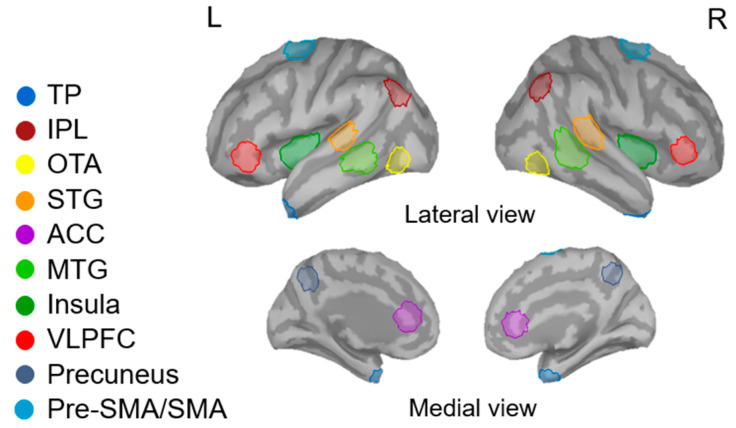
The identified regions of interest (ROIs) on ICBM152 brain template. TP, temporal pole; IPL, inferior parietal lobule; VLPFC, ventrolateral prefrontal cortex; STG, superior temporal gyrus; OTA, occipitotemporal area; MTG, middle temporal gyrus; Pre-SMA/SMA, pre-supplementary motor area and supplementary area; ACC, anterior cingulate cortex; R, right; L, left.

To focus on relevant neural activation in the no-go network, we referred to previous research to identify the functional regions of interest (ROIs) in the cortex. For instance, the inferior frontal gyrus (otherwise known as the ventrolateral prefrontal cortex) is thought to be an inhibition-related region [55,56]. Cortical regions such as the inferior parietal lobule, anterior cingulate cortex, and pre-supplementary motor area are also recruited during no-go processing [57]. Combining the results of grand-averaged source maps and previous research, we identified the following ROIs as involved in response inhibition: bilateral temporal pole (TP), inferior parietal lobule (IPL), ventrolateral prefrontal cortex (VLPFC), occipitotemporal area (OTA), superior temporal gyrus (STG), middle temporal gyrus (MTG), insula, pre-supplementary motor area/supplementary motor area (pre-SMA/SMA), anterior cingulate cortex (ACC), and precuneus (Figure 1). The activity for each source was summarized as the activity of the source with maximal activation for each participant. These ROIs were located in reference to the Desikan-Killiany and Brodmann area templates in the Brainstorm software. For each ROI, the maximal activation cluster was used as the location of the center. A set of 40 vertices corresponding to 5–8 cm^2^ was identified to extract the relative neural amplitudes and latencies in the 200–600 ms time window for further statistical analysis.

In the statistical analysis of each ROI, we examined age-related differences in cortical activation (i.e., peak amplitude and peak latency) in the successful no-go trials using two-factor mixed-design ANOVAs [within-subjects factor: hemisphere (left and right hemispheres); between-subjects factor: age group (younger and older adults)] that were log-transformed to meet the statistical assumptions. Two-way repeated measures ANOVAs (hemisphere × group) were conducted to explore the differences in cortical activation between the high and low performers in the older adult group. The Benjamini and Hochberg procedures [58] were used to correct for multiple comparisons. In accordance with our hypothesis, a one-tailed adjusted *p*-value was used.

## 3. Results

### 3.1. Comparisons between Younger and Older Adult Groups

No significant differences were found between the younger and older adult groups in RT (younger adults = 558.63 ± 12.31 ms, older adults = 554.04 ± 14.72 ms, *p* = 0.853), miss rate (younger adults = 2.09 ± 0.57%, older adults = 1.86 ± 0.56%, *p* = 0.773), or FA rate (younger adults = 6.28 ± 1.70%, older adults = 9.12 ± 1.50%, *p* = 0.216).

**Figure 2:**
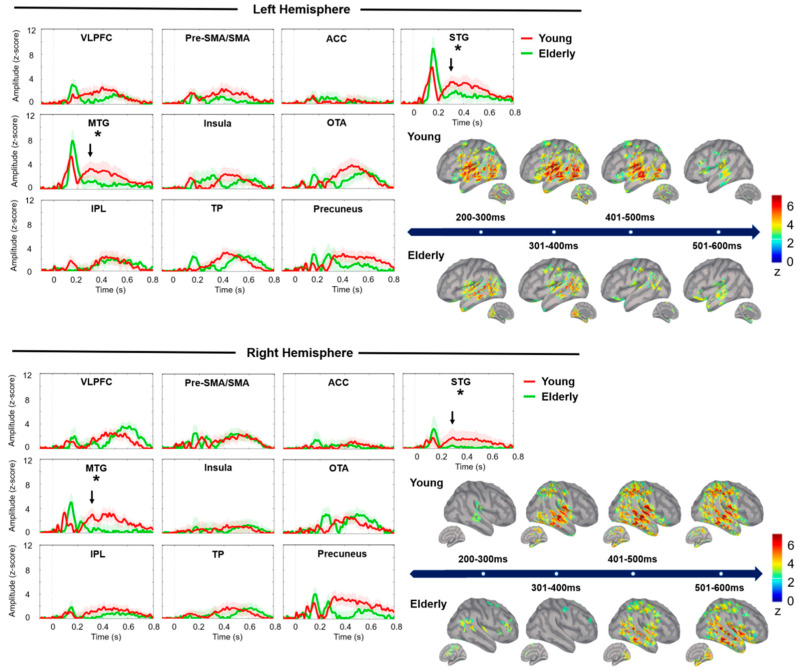
Source waveforms of minimum norm estimate (MNE) are shown from −100 ms to 800 ms in younger (red traces) and older (green traces) adults in each ROI. The stimulus onset is at 0 ms and the shadow of waveforms represents standard errors. Cortical mappings are displayed through the average of cortical activation across every 100-ms time window from 200 to 600 ms corresponding to the temporal processing of response inhibition. Asterisks represent the significant between-group differences in terms of peak amplitudes. STG, superior temporal gyrus; VLPFC, ventrolateral prefrontal cortex; Pre-SMA/SMA, pre-supplementary motor area and supplementary area; TP, temporal pole; ACC, anterior cingulate cortex; MTG, middle temporal gyrus; OTA, occipitotemporal area; IPL, inferior parietal lobule.

The grand-averaged spatiotemporal dynamics of neural responses to successful no-go stimuli between 200 ms and 600 ms are shown in Figure 2. The younger adults exhibited stronger cortical activation in the STG (*F*_1,38_ = 10.01, adjusted *p* = 0.015, partial η^2^ = 0.212) and MTG (*F*_1,38_ = 7.21, adjusted *p* = 0.028, partial η^2^ = 0.146) than the older adults. There were no significant between-group differences in terms of peak latency.

### 3.2. Comparisons between High- and Low-Performing Older Adults

Although the high and low performers were separated based on their FA rate (high performers = 4.68 ± 0.77%, low performers = 13.57 ± 2.13%, *p* = 0.001), these two groups did not differ significantly in terms of RT or miss rate. In addition, there were no significant differences between the high- and low-performing groups in each ROI (i.e., peak amplitude and peak latency) during the MEG recordings. This indicates equivalent cortical activation in both groups.

## 4. Discussion

The current study focused on the effect of age on the cortical activation of response inhibition in an auditory go/no-go task and assessed the differences in cortical activation between high- and low-performing older adults. Our results indicated that the older adults exhibited less cortical activation in the STG and MTG during successful response inhibition. Despite this, we observed no age-related differences in behavioral performance. In addition, we found no significant differences in cortical activation between high- and low-performing older adults in this task.

Our finding that older adults responded competently to specific acoustic cues is supported by previous literature [18,28,29]. There are several lines of evidence showing that the attention of an individual can be modulated by auditory alertness, which facilitates volitional control, leading to the enhancement of behavioral performance [59]. Previous studies have indicated that participants may require more time to identify and respond to auditory cues than to visual ones [34]. This delayed response might reflect the time taken to recapture brief attention lapses and inhibit unnecessary no-go responses [34,60]. Therefore, the auditory alertness and relatively delayed response we speculated may explain the equivalent performance between the younger and older adult groups.

The MEG results revealed diminished cortical activation of the temporal regions (i.e., STG and MTG) in the older adults during successful response inhibition. This finding was consistent with previous studies [18,29], where the age-related potential differences were observed in the midline electrode sites. The auditory ERP activation of the midline electrodes might be the representation of the summation of current activities from bilateral temporal regions. The functional contribution of the STG to inhibition has previously been explored. A study based on go/no-go tasks in awake ferrets suggested that the STG plays an ancillary role in translating the encoding of target stimuli into signals with behavioral meaning, triggering top-down control circuits to initiate goal-oriented behaviors [61]. The neurochemical foundations of the STG might be related to the levels of γ-aminobutyric acid (GABA). The GABA levels in the STG have been reported to predict the behavioral performance of response inhibition [62]. Age-related attenuation of GABA signaling might also influence the neural processing of multisensory integration in audiovisual perception [63,64,65]. Thus, reduced STG activation in the older adults might result in the downregulation of inhibitory processes due to less-efficient integration of sensory information.

The MTG is also thought to contribute to semantic information processing [66]. Dehaene’s model provides a hypothesis relating to the cortical relationships among the STG, the MTG, and the IPL [67,68]. In this model, the STG and MTG are connected through semantically mediated operations in numeral processing [67,68,69]. It has been suggested that the MTG is involved in semantic control related to the interpretation of meanings to guide behaviors [70]. Hence, we suggest that semantic-associated processing may be less efficient in older adults, affecting the detection of target messages that is required to execute context-appropriate behavior [66,71,72].

We found no significant differences in peak latencies, which is inconsistent with previous electrophysiological findings [18,29]. Falkenstein, Hoormann, and Hohnsbein [18] suggested that longer latency arises from slower decision-making processes. As mentioned previously, however, the sensory features of auditory stimuli may alert participants to react [34,59]. If this is the case, a similar peak latency would be expected in younger and older adults, resulting in equivalent RTs. Overall, our findings indicate that auditory tasks are more manageable for older adults than visual tasks to assess cognitive function.

In contrast to our previous findings, we did not find any significant differences between high- and low-performing older adults. Our previous results from a visual go/no-go task indicated that high performers exhibited greater left frontal activation than low performers in an older adult group [22]. The experimental methodology of the two studies is similar apart from the type of stimulus used (visual versus auditory). Hence, we suggest that these two sensory modalities may be differentially affected by age-related degeneration. In addition, the neurological network involved in visual and auditory no-go task processing is likely to be different. For instance, prior studies have demonstrated that the cortical activation of spatial attention and working memory is modality-specific [73,74]. It has also been reported that in early cortico-sensory processing, visual and auditory attentional resources are processed separately [75]. Although there may have been differences in cortical activation between the high and low performers in our auditory go/no-go task, the ROIs we selected did not exhibit relevant differences. Other imaging methods, such as magnetic resonance imaging (MRI), may provide a more detailed, whole-brain analysis of cortical activation.

There were a few limitations in this study. First, spatiotemporally mapped cortical activation using MEG depends on many factors, such as distance from the sensors, the signal-to-noise ratio, the spatial location, and the orientation of the neural currents. For example, the MEG device is more sensitive to tangential currents than radial currents [76], and compared to MRI, MEG sensors are less sensitive in the prefrontal areas and the cerebellum due to limited sensors [35]. These factors might negatively affect the detection of neural activation signals. Second, the ROIs should be identified more carefully. We used templates to identify the ROIs in the cortex of each participant; however, the neural distribution of cognitive processing might vary between individuals. Hence, an investigation involving whole-brain analysis methods, such as MEG or MRI, might provide more detailed results. Finally, the earlier auditory processing (i.e., the 0–200 ms time window after stimulus onset) in the magnetic responses should be taken into consideration in the future investigation on inhibitory function. The period in the 0–200 ms window was thought to reflect the initial sensory and perceptual processing [46,77]. We chose a period of 200–600 ms to examine the effects of age in response inhibition. However, we cannot exclude the earlier auditory processing that might potentially affect the operation of response inhibition.

In conclusion, age-related reduction of cortical activation related to successful response inhibition was observed using an auditory go/no-go task. However, the levels of performance in terms of behavior were similar in younger and older adults. These findings provide evidence of age-related cortical alteration in auditory no-go task processing, thus contributing to future investigations relating to the effects of aging on cognitive activation.
